# Routine blood investigations have limited utility in surveillance of aggressive lymphoma in asymptomatic patients in complete remission

**DOI:** 10.1038/s41416-018-0183-x

**Published:** 2018-07-23

**Authors:** Eliza A Hawkes, Zoe Loh, Ortis Estacio, Geoff Chong, Francis J Ha, Michael Gilbertson, Andrew Grigg

**Affiliations:** 1 Department of Oncology and Clinical Haematology, Austin Hospital, Heidelberg, VIC Australia; 2 Olivia Newton-John Cancer Wellness and Research Centre, Austin Hospital, Heidelberg, VIC Australia; 3 Eastern Health, Box Hill, VIC Australia; 4 University of Melbourne, Parkville, VIC Australia; 5 Monash University, Clayton, VIC Australia; 6 Department of Haematology, Monash Medical Center, Monash Health, Clayton, VIC Australia

**Keywords:** Hodgkin lymphoma, Non-hodgkin lymphoma, Lymphoma

## Abstract

**Background:**

Patients with aggressive lymphoma achieving complete remission (CR) after first-line combination chemotherapy undergo regular surveillance to detect relapse. Current international guidelines recommend routine follow-up blood tests in this context, but evidence supporting this practice is limited.

**Methods:**

We conducted a multi-centre retrospective analysis of all patients diagnosed with aggressive lymphoma treated with curative-intent chemotherapy who achieved CR for at least 3 months between 2000 and 2015. An abnormal blood test was defined as any new and unexplained abnormality for full blood examination, lactate dehydrogenase or erythrocyte sedimentation rate.

**Results:**

Three hundred and forty-six patients attended a total of 3084 outpatient visits; blood tests were performed at 90% of these appointments. Fifty-six (16%) patients relapsed. Routine laboratory testing detected relapse in only three patients (5% of relapses); in the remaining patients, relapse was suspected clinically (80%) or detected by imaging (15%). The sensitivity of all blood tests was 42% and the positive predictive value was 9%. No significant difference in survival was shown in patients who underwent a routine blood test within 3 months prior to relapse versus those who did not (*p* = 0.88).

**Conclusions:**

Routine blood tests demonstrate unacceptably poor performance characteristics, have no impact on survival and thus have limited value in the detection of relapse in routine surveillance.

## Introduction

While the majority of patients with aggressive lymphomas achieve complete remission (CR) with anthracycline-based combination chemotherapy, up to 50% of patients will relapse.^1–3^ As a significant proportion of patients who relapse are considered for salvage chemotherapy and curative-intent autologous stem cell transplant, surveillance after first-line therapy is recommended.^4^

In patients achieving CR, the optimal frequency, duration and type of surveillance are not established. As follow-up imaging is associated with increased radiation-related risk and minimal benefit in asymptomatic patients, such surveillance is no longer routine.^5–7^ Regular laboratory testing (Labs) still features in internationally recognised surveillance guidelines, despite limited evidence for their use in detecting relapse.^7–10^ Studies conducted prior to modern treatment response assessments and routine rituximab administration suggested that lactate dehydrogenase (LDH) and erythrocyte sedimentation rate (ESR) may be useful as surveillance tools, and that, more recently, the absolute lymphocyte count (ALC) and lymphocyte–monocyte ratio (LMR) have shown promise in small series.^11–13^ However, large-scale data are lacking, particularly in the era of positron emission tomography (PET)-defined complete metabolic response (CMR).

Clinically significant scan-related anxiety has been established in both lymphoma and solid malignancies^14,15^; this is reported in up to 80% of patients and does not abate over time. It is likely that blood tests have similar consequences. In addition, routine laboratory investigations have cost implications and are potentially falsely reassuring if normal. Abnormal results are also associated with the potential for expensive, unnecessary additional investigations.

To evaluate the role of routine blood testing in follow-up of patients with aggressive lymphoma, we analysed the use of blood tests in patients with high-grade lymphomas undergoing surveillance after achieving CMR from curative-intent combination chemotherapy at three large Australian cancer centres. In particular, we examined the utility of routine tests for the detection of relapse in the absence of clinical symptoms or signs, and whether performing such tests was associated with significant differences in post-relapse survival.

## Methods

### Patients

Patients were identified from an electronic database at three institutions. Eligible patients were aged 16 years or older, with a documented histological diagnosis of diffuse large B cell lymphoma (DLBCL), Hodgkin's lymphoma (HL), T cell lymphoma (TCL) or Burkitt lymphoma (BL) who received curative-intent first-line treatment and in documented CR on PET/CT for at least 3 months after completion of therapy. Those with primary progressive lymphoma, in partial remission (PR) at the end of first-line treatment, primary central nervous system lymphoma, HIV-associated lymphoma and transformation from indolent subtypes were excluded from the analysis.

All information was obtained from electronic patient records. Data were collected on gender, age, disease stage, comorbidities, presence of B symptoms, Eastern Cooperative Oncology Group performance status, extranodal sites of disease, prognostic score and first-line chemotherapy treatment. Details of each outpatient appointment were recorded, including pathology results, presence of relevant symptoms and/or clinical signs (the absence of both was deemed ‘asymptomatic’), whether the visit was scheduled or unplanned, and outcomes including routine subsequent visit, earlier planned review and results of additional investigations ordered. Relapse date, site and method of diagnosis, any further treatment and date of death or last follow-up were also documented.

Patient follow-up at all three institutions was according to institutional guidelines as follows: 3-monthly for the first 2 years after completion of therapy, and then every 6 to 12 months for the following 3 years for at least 5 years in total. Blood tests were recommended but performed at the treating physician’s discretion. Imaging was also performed according to the treating physician’s discretion but removed from the institutional guidelines in 2014.

### Statistical analysis

The primary endpoints of the study were to assess whether full blood examination (FBE: haemoglobin, white cell count and platelet count), LDH, ESR, ALC, absolute monocyte count (AMC) and LMR during follow-up are reliable markers to predict relapse. Secondary endpoints include methods of relapse detection, event-free survival (EFS) and overall survival (OS). EFS was defined as the period from the date of diagnosis until relapse, disease progression or death from any cause. OS was measured from the date of diagnosis until death from any cause.

Laboratory results were considered abnormal if all of the following were fulfilled: (a) any component of FBE, LDH or ESR fell outside local laboratory normal limits, (b) the derangement was not present previously and (c) could not be explained by a concurrent medical condition. Abnormal laboratory results were investigated at clinician discretion. Laboratory results were evaluated based on their independent ability to detect relapse within 3 months of confirmation. Sensitivity, specificity, negative predictive value (NPV) and positive predictive value (PPV) were derived from 2 × 2 contingency tables and 95% confidence intervals (CIs) were determined exactly.

In addition, receiver operating characteristics (ROC) and area under the curve (AUC) analysis were undertaken to determine the utility of ALC, AMC and LMR as a marker for relapse. AMC and ALC were evaluated as continuous variables, and LMR was calculated by dividing the ALC by the AMC. Survival analysis was performed using the Kaplan–Meier method and compared by the log-rank test between different groups. All values were two-sided and statistical significance was accepted at *p* < 0.05. The study was approved by the local institutional review boards (LR117/2015).

## Results

Between January 2000 and January 2015, 346 eligible patients underwent 3048 outpatient visits. The median follow-up from CR1 was 30 months (range 3–184). Baseline demographics are detailed in Table 1. Laboratory investigations were performed at 2746 visits (90%), with FBE being the most common test ordered (Table 2). LDH was predominantly performed in non-Hodgkin's lymphoma (NHL) and ESR in HL.

**Table 1:** Baseline demographics

	All patients *n* = 346 (%)
Median age (range)	54 (17–91)
Gender	
Male	202 (58)
Female	144 (42)
Stage	
I–II	168 (49)
II–IV	178 (51)
Subtype	
DLBCL	187 (54)
HL	119 (34)
BL	22 (6)
TCL	18 (5)
B symptoms	201(58)
>1 extranodal site	68 (20)
Bone marrow involvement	39 (11)
Performance status	
0–1	306 (88)
≥2	40 (12)
Prognostic score	
Low risk^a^	280 (81)
High risk^b^	66 (19)
Treatment	
Chemotherapy alone	238 (69)
Chemotherapy and radiotherapy	108 (31)

^a^Low risk = International Prognostic Index for DLBCL and TCL: 0–2; Hasenclever score for HL: 0–3; prognostic score for Burkitt’s lymphoma: 0–2.

^b^High risk = International Prognostic Index for DLBCL and TCL: >3; Hasenclever score for HL: >4; prognostic score for Burkitt’s lymphoma: >3

**Table 2:** Laboratory results

	All visits (*n* = 3048)		NHL (*n* = 1908)		HL (*n* = 1140)	
	Total	Abnormal	Total	Abnormal	Total	Abnormal
Any labs	2746	404 (15%)	1707	279 (16%)	1039	125 (12%)
FBE	2660	271 (10%)	1638	195 (12%)	1022	76 (7%)
LDH	2147	187 (9%)	1362	135 (10%)	785	52 (7%)
ESR	411	25 (6%)	—	—	406	25 (6%)

Relapse of lymphoma occurred in 56/346 (16%) patients (33 DLBCL, 19 HL, 4 other). The median age at relapse was 64.3 years (range 18–91), and 51% were over 60 years of age. Forty-three out of 56 (77%) had advanced stage disease and 18/56 (32%) were at high risk (as defined in Table 1). Only one patient (high-risk HL) received an abbreviated chemotherapy course; the remaining 45 patients received a full course of standard treatment. The median duration from treatment completion until relapse was 14 months (range 3–84 months), with 48% of relapses occurring in the first year, 31% in the second year and the remainder (21%) occurring up to 7 years after the end of treatment.

Relapse was diagnosed by routine laboratory investigations in 3/56 (5%) and routine imaging in 10/56 (18%) patients. Clinical symptoms/signs lead to diagnosis of relapse in 43/56 (80%; 40 with symptoms, 3 with signs only); 19 of which were detected at unscheduled visits. Unscheduled appointments due to patient-reported symptoms (3% of all visits) showed a significantly stronger association with relapse than scheduled visits (odds ratio 50.4, *p* < 0.001).

Abnormal laboratory results were recorded at 404/3048 follow-up visits: 304 in asymptomatic and 100 in symptomatic patients.

### Asymptomatic patients

An unexplained abnormal result prompted a change in management at 46/304 (15%) visits in asymptomatic patients: 19/46 (41%) had repeat interval laboratory investigations only, 13/46 (28%) underwent additional imaging, 10/46 (22%) were booked for an earlier future review with repeat labs and 4/46 (9%) had biopsies in addition to imaging. The specific laboratory abnormalities and associated changes in management in asymptomatic patients at scheduled appointments are described in Table 3. Almost all elevations in LDH and ESR were <2 times the upper limit of normal (ULN), and leukopaenia was the most common FBE abnormality (12/29; 41%) resulting in change in management.

**Table 3:** Abnormal laboratory results in asymptomatic patients at scheduled appointments

	Asymptomatic		
	FBE (all patients; *n* = 2191)	LDH (NHL; *n* = 1095)	ESR (HL; *n* = 343)
No. abnormal results	190/2191 (9%)	99/1095 (9%)	17/1343 (5%)
Degree of abnormality			
<2 × ULN	N/A	98 (99%)	16 (94%)
>2 × ULN		1 (1%)	1 (6%)
Change in management:	29/190 (15%)	16/99 (16%)	3/17 (18%)
Earlier subsequent review	6	2	0
Additional lab tests	16	10	2
Imaging	4	3	1
Biopsy	3	1	0
Specific abnormality resulting in change in management	Leukopaenia 13	<2 × ULN: 15	<2 × ULN: 3
	Leukocytosis 4	>2 × ULN: 1	
	Anaemia 4		
	Thrombocytopaenia 4		
	Pancytopaenia 3		
	Thrombocytosis 1		
Relapse detected due to further investigation of labs	0	1	1
Relapse within 3 months of abnormality^a^	5	4	1

^a^Including cases where relapse was diagnosed only after symptoms developed

Relapse was diagnosed by 3/304 (1%) abnormal results in asymptomatic patients; one TCL with neutropaenia and thrombocytopaenia, and two HL patients; one with elevated LDH and one with elevated ESR. No relapses in NHL were diagnosed on the basis of an abnormal LDH alone.

In five additional patients, relapse was detected within 3 months of an abnormal result; however, in these cases, suspicion of relapse arose only after the patient developed symptoms. The abnormalities were: lymphopaenia, elevated LDH and both elevated LDH and abnormal FBE in three patients.

### Symptomatic patients

In contrast, 67/100 (67%) of symptomatic patients with an abnormal result underwent a change in their management. The most common changes were further imaging (*n* = 35; 52%) and imaging with biopsy (*n* = 17; 25%), followed by earlier future review and labs (*n* = 7; 10%) and repeat interval laboratory investigations only (*n* = 8;12%).

The sensitivity, specificity, PPV and NPV of all routine lab tests (FBE, LDH and ESR combined) in detecting relapse was 42%, 87%, 9% and 98%, respectively. Performance characteristics of individual lab tests are detailed in Table 4. The PPV of LDH remained the same even in the subset of 115 NHL patients with elevated baseline LDH (8%, confidence interval (CI), 5–12). In the 43 HL patients with an elevated baseline ESR, the PPV of ESR was even lower (6.5%, CI, 2–17).

**Table 4:** Performance of testing

	All tests		FBE		LDH (NHL)		ESR (HL)	
Sensitivity	0.42	(31–52)^a^	0.46	(35–57)	0.28	(15–44)	0.39	(17–64)
Specificity	0.87	(86–89)	0.75	(73–76)	0.89	(87–90)	0.86	(82–89)
PPV	0.09	(7–13)	0.05	(4–7)	0.08	(4–13)	0.11	(5–22)
NPV	0.98	(87–93)	0.98	(97–98)	0.97	(96–98)	0.97	(94–99)

*PPV* positive predictive value, *NPV* negative predictive value.

^a^ 95% confidence interval

ROC and AUC analysis showed that ALC, AMC and LMR at each appointment (*n* = 2660) were all very poor markers for relapse (AUC = 0.517, 0.529 and 0.577 respectively); thus, their performance characteristics were not calculated.

**Fig. 1:**
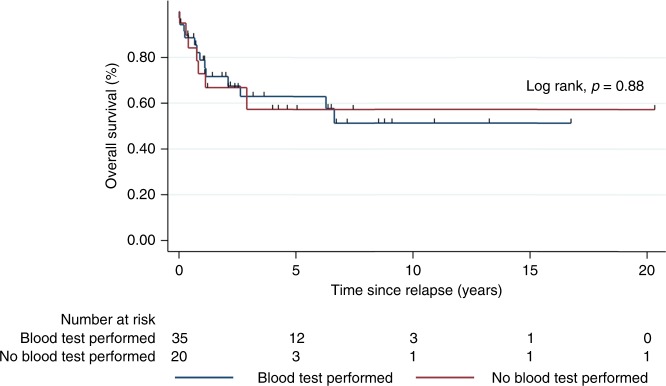
Survival curve in relapsed patients. This shows the post-relapse survival based on whether a blood test was performed in preclinical period (3 months) for relapse

Two-year OS and EFS were 76% (95% CI, 71–80) and 70% (95% CI, 65–80), respectively, in the whole cohort. There was no significant difference in post-relapse survival between patients who had laboratory investigations performed ≤3 months prior to documented relapse versus patients who did not (*p* = 0.88, Fig. 1).

## Discussion

This analysis, from one of the largest and most comprehensive series in the modern era to our knowledge, demonstrates little benefit of including routine laboratory testing to detect relapse in follow-up of asymptomatic patients with aggressive lymphoma achieving metabolic CR after first-line chemotherapy. In line with published reports, our results confirm that clinical symptoms and signs are the single most important predictor of relapse,^16,17^ with 80% of relapsed patients having symptoms at presentation and only 1% of isolated abnormal blood test results leading to a diagnosis of relapse. There was no difference in survival between patients who had blood tests and those who did not.

Previous studies have reported that routine blood tests do not reliably predict relapse.^17–19^ However, all have assessed either only one parameter or ‘blood tests’ as a whole without describing which tests were performed or omitted. Our study is the only one to assess the performance of individual tests, their role in the detection of relapse and their impact on management and overall outcomes in a population with PET-confirmed CR at 3 months.

ESR had been proposed as a useful marker of relapse in HL in 1991^20^, but subsequent studies dispute this, with the vast majority of relapses detected by clinical findings rather than by ESR alone.^21,22^ Nevertheless, ESR is still frequently performed during follow-up. In our cohort, ESR had a sensitivity of only 39% for detection of relapse in HL, and only one relapse was diagnosed by an isolated elevated ESR.

LDH has also been proposed as a useful screening test for relapse in DLBCL in the pre-rituximab era^23^, but recent studies are consistent with ours in showing its lack of predictive value in the absence of symptoms or signs suggesting relapse.^13,18,24,25^ The PPV of an elevated LDH in our aggressive NHL cohort was 8%, even after accounting for known causes of LDH elevation such as liver disease and infection. No relapses in NHL were detected on the basis of LDH alone. Our findings confirm results from a previous smaller series of 100 DLBCL patients,^19^ which analysed LDH at every appointment and reported a low PPV of 9% and sensitivity of 47% for relapse. Interestingly, LDH was ordered at 69% of HL follow-up appointments, despite a lack of evidence or recommendations by guidelines for its use in monitoring this subtype, and lead to the detection of one HL relapse.

FBE was the most commonly performed test in our study, with an abnormal result in 10% of samples, yet was associated with a change in management in <15% of the time. There was one relapse diagnosed on the basis of FBE alone. These findings are also consistent with the literature, with several studies reporting no relapses detected by FBE abnormalities.^21–23^

Baseline lymphocyte and monocyte counts and the LMR have prognostic value for both DLBCL^26^ and HL^27^ and three retrospective studies concluded that a low ALC and LMR during follow-up is a useful indicator of relapse in DLBCL. PPV and NPV in these studies ranged between 68–74% and 49–96%, respectively, with sensitivity 68–89% and specificity 88%.^11–13^ However, these studies analysed parameters at a single time point just prior to relapse without accounting for symptomatology or confirming initial CR on PET. In contrast, our analysis demonstrated that ALC, AMC and LMR had almost no ability to discriminate between relapsed and non-relapsed patients, with far lower AUC values than previously reported (0.52 versus 0.91 for ALC).^13^

It may be argued that the NPV of laboratory tests was high in our study (98%) and provides reassurance to patients with normal results. Conversely, 15% of blood tests had an unexplained abnormality; not only are they of poor PPV in asymptomatic patients, they almost always result in unnecessary patient anxiety and often lead to further investigations, which are seldom abnormal. Routine blood tests have been postulated as a method of monitoring for therapy-related myelodysplastic syndromes (MDS). The incidence of therapy-related MDS in patients receiving induction chemotherapy for aggressive lymphomas is only marginally higher than the general population (0.4–1.2% post treatment versus 0.3% in the general population^28–30^). More importantly, there is limited evidence for early detection of MDS in asymptomatic patients and current guidelines do not recommend treatment for the majority of this cohort. Additionally, screening for MDS would, at most, warrant a FBE alone, but not other currently recommended blood tests in lymphoma surveillance guidelines.

Recognising this study is retrospective, and the design remains robust. The patients were treated uniformly, as demonstrated by the high percentage of patients undergoing the individual blood tests, consistent use of end of treatment PET to confirm metabolic remission and limited variation in treatment regimens. Unlike the majority of prior analyses,^17–19^ this study reviewed all labs performed for the duration of follow-up in patients with PET-confirmed CR for at least 3 months following treatment. Our study included all major histological subtypes of aggressive lymphoma and is likely relevant to a wider population. Although of note, the exclusion of primary refractory disease in our cohort to accurately analyse the role of blood tests in detection of relapse led to a lower proportion of high-risk patients than many published series.

This study confirms that common blood tests do not reliably detect relapse of aggressive lymphoma in asymptomatic patients treated in the modern era and should not be recommended by current international guidelines. They are no longer performed in this context in our institutions. More novel methods of relapse detection such as circulating tumour DNA have demonstrated greater specificity and sensitivity than standard blood parameters; however, this technology is yet to be widely available and affordable.

## Availability of data and material

Materials, data and associated protocols are available from the corresponding author on reasonable request.
